# Evaluation of the effectiveness of nutrition education program in promoting healthy dietary habits in Memphis, Tennessee: a quasi-experimental pilot study

**DOI:** 10.1186/s12889-026-27392-3

**Published:** 2026-04-14

**Authors:** Edmore Madondo, Sharon Moore, Gabriella Huffstetler, Elaina Kaufman, Butch Odom, Kimberly Boone, Fawaz Mzayek, Debra Bartelli

**Affiliations:** 1 Division of Epidemiology, Biostatistics & Environmental Health, School of Public Health, The University of Memphis, Memphis, Tennessee USA; 2 Church Health, Memphis, Tennessee USA

**Keywords:** Cooking confidence, Mediterranean diet, Life balance

## Abstract

**Background:**

Poor diet is a major public health concern due to its association with chronic diseases. Church Health, a not-for-profit and faith-based healthcare organization in Memphis, Tennessee, implemented Cook Well, Be Well (CWBW), a 4-session nutrition education program designed to improve dietary habits through instruction on the Mediterranean diet (MD), the Model for Healthy Living (MFHL), and healthy cooking. This study evaluated the program’s effectiveness in promoting healthier eating behaviors and cooking confidence.

**Methods:**

Secondary data from pre- and post-intervention surveys were analyzed. CWBW comprises four weekly sessions for individuals aged ≥ 16 years. Outcomes included changes in adherence to the MD and MFHL and cooking confidence, assessed using items from the CWBW questionnaire (scored 0–2). The MD section had nine questions, the MFHL had seven, and the cooking confidence section included 11. A multivariable linear mixed-effect regression model was used to examine the effect of CWBW participation on each outcome, adjusting for baseline measurements and sociodemographic variables. The Wilcoxon Signed Rank test was used to examine differences across various sociodemographic characteristics. For each outcome, mean or median differences, 95% confidence intervals (CI), *p*-values, and effect sizes (Cohen’s *d*) were reported.

**Results:**

Data from 351 participants were analyzed (52.4% female, 27.6% African American). The overall score (sum of MD, MFHL, and cooking confidence) increased significantly (mean difference [md] = 4.72; 95% CI: 2.93, 6.50, *P* < .01, *d* = 0.72). Significant increases were observed in MD scores (md = 0.96; 95% CI: 0.35, 1.57, *P* = .01, *d* = 0.43), cooking confidence (md = 2.27; 95% CI: 1.23, 3.32, *P* < .01, *d* = 0.59), and MFHL scores (md = 1.48; 95% CI: 0.79, 2.17, *P* < .01, *d* = 0.59). These improvements were sustained at 3-, 6-, and 12 months for MD and cooking confidence (*P* < .05).

**Conclusion:**

The CWBW program significantly improved participants’ adherence to the MD, confidence in healthy cooking, and engagement with the MFHL. These findings suggest that community-based nutrition education programs, particularly when rooted in cultural and faith-based contexts, can effectively promote healthier dietary behaviors and support chronic disease prevention.

**Supplementary Information:**

The online version contains supplementary material available at 10.1186/s12889-026-27392-3.

## Background

An unhealthy diet is a significant public health challenge because of its association with obesity and other diet-related chronic diseases, including cardiovascular diseases (CVDs), some types of cancer, and type 2 diabetes [1]. Chronic diseases are the leading cause of mortality and morbidity among the United States (US) adult population [2]. In Shelby County, Tennessee, the age-adjusted prevalence of diagnosed diabetes is estimated to be 12.0% [3], and it is among the county’s ten leading causes of death (29 per 100,000 people) together with heart diseases (178 per 100,000 people) [4].

According to the 2020–2025 Dietary Guidelines for Americans (DGA), a healthy diet should include vegetables, fruits, whole grains, and protein-rich food such as fish and should limit the consumption of refined and processed foods [5]. The DGA promotes the Mediterranean diet (MD) as a healthy dietary pattern which emphasizes plant-based foods, unsaturated fats, fish oils, and low consumption of dairy, meat, and alcohol [6]. Multiple studies have demonstrated that the MD reduces body weight, improves insulin sensitivity, lowers HbA1c levels, and decreases cardiovascular risk factors in individuals with or at risk for type 2 diabetes [7–9]. However, at least 80% of Americans eat food that is low in vegetables, fruits, and dairy [5]. Factors that contribute to poor dietary patterns include financial constraints [10], limited nutrition knowledge, and insufficient cooking skills [11].

One of the best ways to improve cooking confidence and nutrition knowledge is by implementing nutrition education programs [12]. For instance, researchers in Brazil who implemented a pilot program, Nutrition and Culinary in the Kitchen (NCK), reported a 45.37% ± 93.57% increase in culinary skills among individuals in the intervention group [13]. Similarly, a pre-test/post-test cooking program conducted in Leeds, UK reported a significant increase in cooking confidence by 1.7 (95% CI: 1.6, 1.9, *P* < .001), along with an increase in self-reported daily intake of fruits and vegetables by 1.5 (95% CI: 1.3, 1.6, *P* < .001) [14]. Further supporting the benefits of such programs, a cross-sectional study of 185 adults with overweight or obesity, by Arslan et al. examined the relationship between food and cooking skills, cooking confidence, and eating behaviors [15]. Using validated questionnaires, the authors found that higher food and cooking skills, as well as greater cooking confidence, were positively associated with cognitive restraint, emotional eating, and uncontrolled eating, after adjusting for sociodemographic and health factors [15]. These results suggest that improving cooking confidence and practical cooking skills may enhance eating awareness and support healthier dietary behaviors in this population.

To encourage the practice of healthy eating, Church Health, a not-for-profit and faith-based healthcare organization in Memphis, Tennessee, developed and implemented a four-week pilot nutrition and cooking education program, Cook Well, Be Well (CWBW). The program focused on educating participants about the principles of the MD, healthy cooking techniques to prevent and manage chronic diseases, and the Model for Healthy Living (MFHL), a life balance tool created by Church Health that is based on the belief that health is not just about physical wellness but involves the balancing of seven dimensions of life: faith life, medical, movement, work, emotional, nutrition, and friends and family. The two-hour weekly sessions included a nutrition presentation covering principles of the MD and the MFHL, nutrition information, and cooking demonstrations featuring recipes suited to the MD. Classes for the pilot program ran from February 2021 to September 2023. The objectives of the program were for participants to become more comfortable in the kitchen, build confidence in preparing basic healthy meals, increase the consumption of fruits, vegetables, grains, and healthy fats, and cook on a budget. At the conclusion of the pilot program, investigators at the University of Memphis were asked to evaluate the program’s effectiveness. This study, therefore, reports the results of secondary data analysis of CWBW.

This study advances the literature by not only confirming that community-based nutrition and cooking programs can improve dietary habits and cooking confidence, but also by quantifying effect sizes, demonstrating effectiveness across diverse populations, and integrating a holistic life balance model into such programs. It provides practical, scalable evidence for implementing such programs in high-burden, underserved areas. The objective of the study was to evaluate the effectiveness and the magnitude of CWBW program’s impact in promoting healthy dietary habits. We hypothesized that providing education about nutrition and cooking skills would improve healthy dietary habits of the program participants.

## Methods

### Study area

Church Health is located in Memphis, Tennessee. With an estimated population of 633,104 [16], Memphis is the second largest city in the State of Tennessee. Only 28.6% of the total population has attained at least a Bachelors’ degree and 13.8% lack any form of health care coverage [16].

### Study design

This study used a quasi-experimental, one-group pre-post design to evaluate the effectiveness of CWBW, a community-based nutrition education and cooking program for individuals ≥ 16 years of age. The program was piloted by Church Health, in Memphis, Tennessee from February 2021 to September 2023. In this program, participants were invited to attend one two-hour session of CWBW per week for four weeks, either in-person or virtually. Classes were offered in Spanish and English. Participants were taught the principles of both the MD and the MFHL, as well as basic cooking skills. Participants were provided with a combination of handouts (e.g., recipes, cooking tools, food safety, portion control, and cooking on a budget) presentations about the principles of the MD and the MFHL by the organization’s registered dietitians, and demonstrations of cooking techniques such as knife skills from professional cooking instructors. In-person participants received hands-on cooking experience during class while those who attend classes virtually cooked from their home. Both in-person and online participants were able to sample the food that was prepared. Details about the CWBW program and class schedule are found at: https://churchhealth.org/cook-well-be-well/.

### Study population

**Fig. 1:**
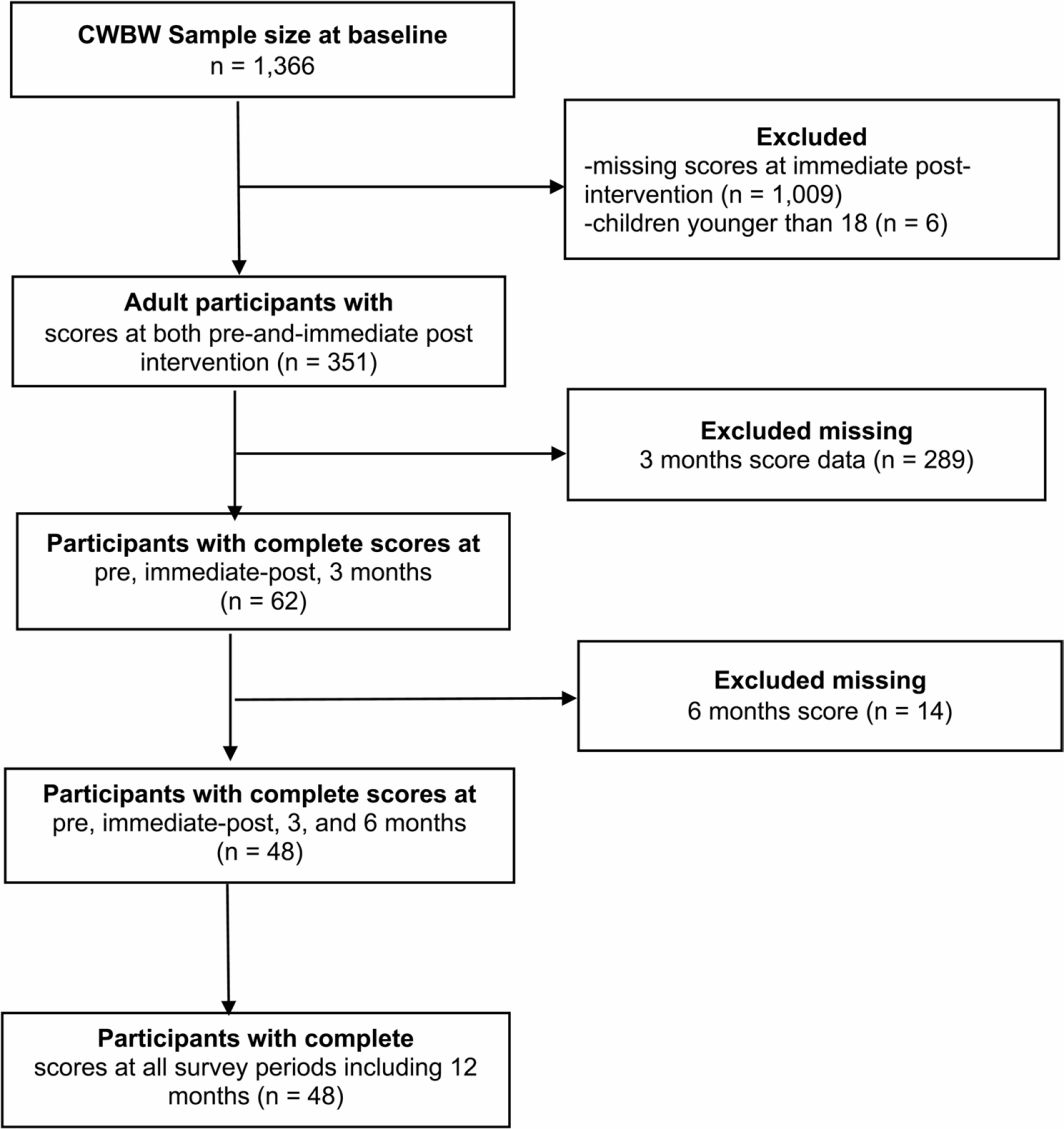
Flow chart of participants’ selection criteria

The pilot CWBW program had a total of 1,366 participants, of which 1,009 were excluded because of missing data on the main outcomes at immediate-post intervention (Fig. 1). We also excluded participants younger than 18 years (*n* = 6). The main analysis focused on data collected at pre- and-immediate post intervention (*n* = 351). However, the results for data collected in post-intervention months 3-, 6-, and 12 are also provided (Table 3).

### Participant recruitment

To maximize participant enrolment into the program, Church Health employed various recruitment approaches including promotion among their own patient population and that of other local health care organizations and academic institutions, which helped spread the message about the CWBW program to the Memphis community. Other approaches were designed specifically to target diverse populations and these included video commercials in health clinic waiting rooms and social media posts. Enrolment in the program was free. However, the virtual class participants were required to purchase their food ingredients for the cooking sessions. This study was conducted following the ethical guidelines outlined in the Declaration of Helsinki and was approved by the University of Memphis IRB (ID: PRO-FY2025-83). Before data collection, the consent to participate in the study was sought from all participants. All data were de-identified by Church Health.

### Study instruments and measures

All participants in the CWBW program were invited by program staff to complete an online or paper-based questionnaire at pre-intervention, immediate post intervention, and at three (3) months, six (6) months, and twelve (12) months post-intervention. The questionnaire was available in both English and Spanish. Except for the pre-intervention survey, participants were not required to complete the surveys, and no incentives were provided for survey completion. Participant sociodemographic and health status data were collected at pre-intervention only. Knowledge of MD and MFHL principles, cooking skills, and dietary practices were assessed at all time points. The MD [17, 18] and cooking skills questions [19, 20] were adapted from validated instruments used in previous nutrition-related intervention studies. The MFHL questions have not been validated but were based on previous studies that found associations between factors related to balance life (time constraints, exercise, and stress) and healthy eating patterns [21, 22] and further developed by Church Health in 2021 to suit CWBW program objectives. The final questionnaire is found in CWBW Supplemental File (Supplemental Material 1). The main outcomes of interest for this study were adherence to the MD and MFHL, and cooking confidence scores which were calculated using a set of items from the CWBW program questionnaire. The MD section contained nine (9) questions (e.g. How many cups of vegetables do you consume each day? ), scored using a scale ranging from 0 (< 0.5 cups/day) to 2 (> 1 cup/day). The cooking confidence section had eleven (11) questions (e.g. How confident do you feel about being able to cook from basic ingredients? ) scored from 0 (Not at all confident) to 2 (Very confident). The MFHL section had seven (7) questions (e.g. How frequently do you engage in physical activity each week? ) scored from 0 (0–2 days) to 2 (5–7 days). The criteria for scoring MD, MFHL and cooking skills were adapted from previous studies [17, 19] (see Table S1). The maximum possible points for the MD, MFHL, and cooking confidence were 18, 14, and 22, respectively. Higher scores indicate a higher level of cooking confidence and adherence to the MD and MFHL principles.

### Statistical analysis

We summarized baseline characteristics using frequency and percentages (%). A multivariable linear mixed-effects model was used to examine the effect of CWBW participation on each outcome, adjusting for age, gender, education, marital status, race, income level, education, employment status, health conditions, and class type (in-person vs. online). The Wilcoxon Signed Rank Test was used to test whether the median pre- and post-intervention scores are different. For parametric data, mean difference of the scores and 95% confidence interval (CI) were reported; while for non-parametric data, the median difference and interquartile range (IQR) were reported. The effect size of the intervention was estimated by Cohen’s *d* [23]. We categorized the effect size as follows: small (*d* = 0.2), moderate (*d* = 0.5), and large (*d* ≥ 0.8) [24]. We performed subgroup analysis to test whether the effect of the CWBW program on cooking confidence, and adherence to the MD and MFHL differed across various sociodemographic characteristics. To determine if there was a statistically significant proportional difference from pre- to immediate post-intervention, a McNemar-Bowker test was used. A *P <* .05 was considered statistically significant. For data analysis, we used SAS version 9.4 (SAS Institute Inc., Cary, NC, USA).

## Results

### Baseline characteristics of CWBW program participants

Table 1 shows the baseline characteristics of individuals enrolled in the CWBW nutrition education and cooking class program. Among the participants with available information a larger proportion were women, single, African American, and attended classes in person.

**Table 1:** Baseline characteristics of CWBW cooking class program participants (*n* = 351)

Variable	Number of participants	Percentage (%)
Age (years)		
18–44	115	32.8
> 44	95	27.1
Missing (40.1%)		
Gender		
Male	26	7.4
Female	184	52.4
Missing (40.2%)		
Marital status		
Married	89	25.4
Single	121	34.5
Missing (34.1%)		
Race		
African American	97	27.6
White/Caucasian	50	14.2
Other	63	17.9
Missing (34.1%)		
Hispanic		
Yes	70	19.9
No	140	39.9
Missing (40.1%)		
Income level, $		
<=20,000	209	59.5
20,001–50,000	83	23.6
50,001 +	59	16.8
Education		
College	142	40.5
High School	48	13.7
Less than High School	20	5.7
Missing (40.1%)		
Employment status		
Employed	42	12.0
Unemployed	64	18.2
Missing (69.8%)		
Health conditions		
Hypertension	40	11.4
Prediabetes/Diabetes	48	13.7
Other Chronic Diseases	26	7.4
None of these	96	27.4
Missing (40.1%)		
Class Type		
In-person	323	92.0
Online	28	8.0

### Effect of CWBW program on participants’ healthy dietary habits

Table 2 shows the effect of the CWBW nutrition education and cooking class program on participants’ healthy eating habits. On average, the overall scores increased significantly by 4.72 points (95% CI: 2.93, 6.50, *P* < .01; *d* = 0.72), MD score improved significantly by 0.96 points (95% CI: 0.35, 1.57, *P* = .01; *d* = 0.43) from the baseline to immediate post-intervention. Specifically, more participants reported consuming more than 1 cup of vegetables, fruits and nuts per day; more than two servings of fish per week, olive oil, and reduction in the consumption of more than 7 ounces of cereals at immediate post intervention (Table S2). The mean score for cooking skills confidence increased by 2.27 points (95% CI: 1.23, 3.32, *P* < .01; *d* = 0.59). Among the elements of the cooking confidence skills, more participants reported being very confident in cooking from basic ingredients, following a simple recipe, preparing and cooking, and tasting new foods. Additionally, more participants reported making substitutions for items in a recipe, using spices/herbs, handling a knife safely, eating beans, chickpeas, and meat alternatives once/week, and using leftovers to reduce consumption of convenient foods and ready-made meals (Table S3). The mean difference score for MFHL is 1.48 (95% CI: 0.79, 2.17, *P* < .01; *d* = 0.59). More specifically, the proportion of participants who made smart food choices 5–7 days/week; engaged in physical activity 3–5 days/week; managed stress and understand feelings most days; received support through relationships most days and managed their medical care most of the time increased (Table S4).

**Table 2:** Effect of CWBW on participants’ healthy dietary habits: pre- to-immediate post (*n* = 351)

Variable	Model I			Model II		
	md [95%CI]	*P*	Cohen’s d	md [95%CI]	*P*	Cohen’s d
Mediterranean Diet score	1.12 [0.74–1.49]	**< 0.01**	0.44	0.96 [0.35–1.57]	**0.01**	0.43
Cooking Confidence score	2.56 [2.01–3.11]	**< 0.01**	0.69	2.27 [1.23–3.32]	**< 0.01**	0.59
Model for Healthy Living Score	1.40 [1.02–1.77]	**< 0.01**	0.55	1.48 [0.79–2.17]	**< 0.01**	0.59
Overall score	5.07 [4.11–6.03]	**< 0.01**	0.78	4.72 [2.93–6.50]	**< 0.01**	0.72

*md* mean difference, *CI* confidence interval, *Model I* no covariate was adjusted, *Model II* adjusted for age, gender, education, marital status, race, income, education, education, employment status, health conditions, and class type

*P* < .05 was considered statistically significant. *P* in bold means the result is statistically significant

In Table 3, the results show that there was a statistically significant increase in the median scores for adherence to the MD, cooking confidence skills, and overall scores at 3-, 6-, and 12- months post intervention (*P* < .05). For the MFHL, a significant increase in the median scores was only observed at 3- and 12- months post-intervention (*P* < .05).

**Table 3:** Effect of CWBW on participants’ health dietary habits

Variable	Baseline − 3 months (*n* = 62)		Baseline − 6months (*n* = 48)		Baseline – 12 months (*n* = 48)	
	Median difference [IQR]	*P*	Median difference [IQR]	*P*	Median difference [IQR]	*P*
Mediterranean Diet Score	2.0 [1.0–4.0]	**< 0.01**	2.0 [0.0–4.0]	**< 0.01**	1.0 [0.0–4.0]	**0.01**
Cooking Confidence Score	3.0 [0.0–6.0]	**< 0.01**	4.0 [1.0–7.5]	**< 0.01**	4.0 [0.0–9.0]	**0.01**
Model for Healthy Living Score	1.0 [-1.0–4.0]	**0.01**	0.5 [− 3.0–4.0]	0.4	1.0 [-1.0–3.0]	**0.01**
Overall Score	7.0 [2.0–14.0]	**< 0.01**	7.5 [-0.5–15.0]	**< 0.01**	7.0 [− 3.0–13.0]	**0.01**

Wilcoxon Signed Rank test was used to test the differences in median

*IQR* interquartile range

A *P* < .05 was considered statistically significant. *P* in bold means the result is statistically significant

### Effect of CWBW on healthy dietary habits of participants in different subpopulations

Table 4 shows score changes from pre- to immediate post-intervention across various sociodemographic characteristics. Across all racial groups and marital status, there were statistically significant improvements in median MD, cooking confidence, MFHL, and overall scores (*P* < .05). Among females, participants aged 18–44, employed, individuals with annual incomes up to $50,000, and those who attained at least high school, all four scores increased significantly. In contrast, participants older than 44 years experienced significant increases only in MD, cooking confidence, and overall scores. Among participants earning $50,000 or more, only cooking confidence and overall scores improved significantly. For those attending classes in person, significant increases were observed in all outcomes. In contrast, among those attending only online, MD scores did not increase significantly.

**Table 4:** Changes in scores between pre-and immediate-post intervention in different subgroups

Variable	*N*	MD Scores		Cooking Confidence Scores		MFHL Scores		Overall Score	
		Median (IQR)	*P*	Median (IQR)	*P*	Median (IQR)	*P*	Median (IQR)	*P*
Age, years									
18–44	115	2.0 (-1.0–4.0)	**< 0.01**	3.0 (-1.0–7.0)	**< 0.01**	2.0 (0.0–4.0)	**< 0.01**	6.0 (0.0–12.0)	**< 0.01**
45+	95	1.0 (-2.0–3.0)	**0.03**	2.0 (-2.0–6.0)	**0.01**	0.0 (-2.0–3.0)	0.09	3.0 (-2.0–10.0)	**0.01**
Gender									
Male	26	2.0 (-1.0–3.0)	0.20	1.5 (0.0–5.0)	**0.02**	0.5 (-2.0–3.0)	0.36	3.0 (-2.0–10.0)	**0.02**
Female	184	1.0 (-1.0–3.5)	**< 0.01**	3.0 (-1.0–7.0)	**< 0.01**	1.0 (-1.0–4.0)	**< 0.01**	5.0 (0.0–11.0)	**< 0.01**
Education									
College	142	1.0 (-1.0–3.0)	**0.01**	2.0 (-1.0–6.0)	**< 0.01**	1.0 (-2.0–3.0)	**0.01**	5.0 (-1.0–11.0)	**< 0.01**
High School	48	1.0 (-1.0–4.0)	**0.01**	3.0 (-0.5–7.0)	**< 0.01**	2.0 (0.0–4.0)	**< 0.01**	8.0 (-0.5–11.0)	**< 0.01**
< High School	20	1.0 (-1.0–4.0)	**0.05**	2.0 (-0.5–6.0)	**0.07**	-0.5 (1.0–4.5)	**0.02**	4.5 (0.5–10.5)	**0.01**
Race									
African American	97	1.0 (-2.0–3.0)	**0.02**	3.0 (-1.0–7.0)	**< 0.01**	1.0 (-2.0–3.0)	**0.10**	5.0 (-2.0–11.0)	**< 0.01**
White/Caucasian	50	2.0 (-2.0–4.0)	**0.03**	1.0 (-1.0–6.0)	**0.01**	1.0 (-1.0–4.0)	**0.01**	3.5 (-2.0–11.0)	**0.01**
Other	63	1.0 (-1.0–4.0)	**< 0.01**	2.0 (-1.0–7.0)	**0.01**	3.0 (0.0–5.0)	**< 0.01**	0.05 (1.0–12.0)	**< 0.01**
Employed									
Yes	42	1.5 (-1.0–3.0)	**0.01**	3.0 (0.0–7.0)	**0.01**	3.0 (1.0–5.0)	**< 0.01**	8.5 (3.0–8.5)	**< 0.01**
No	64	0.5 (-1.0–3.0)	**0.08**	2.0 (-2.0–6.0)	**0.01**	0.5 (-2.0–3.0)	**0.18**	3.0 (-1.5–9.0)	**0.01**
Income									
<=20,000	209	1.0 (-2.0–3.0)	**< 0.01**	3.0 (-1.0–7.0)	**< 0.01**	1.0 (-1.0–4.0)	**< 0.01**	5.0 (-1.0–12.0)	**< 0.01**
20,001–50,000	83	2.0 (-1.0–4.0)	**< 0.01**	3.0 (-1.0–7.0)	**< 0.01**	2.0 (0.0–4.0)	**< 0.01**	7.0 (0.0–13.0)	**< 0.01**
50,001 +	59	0.0 (-2.0–3.0)	0.26	2.0 (-1.0–6.0)	**0.01**	-1.0 (-3.0–2.0)	0.69	3.0 (-3.0–8.0)	**0.01**
Marital Status*									
Married	89	1.0 (0.3–1.8)	**.0**1	2.0 (1.0–3.1)	**0.01**	0.9 (0.2–1.6)	**0.01**	4.0 (2.3–5.6)	**< 0.01**
Single	121	1.3 (0.6–2.0)	**0.01**	2.9 (1.9–3.8)	**< 0.01**	1.6 (1.0–2.3)	**< 0.01**	5.8 (4.1– 7.5)	**< 0.01**
Class delivery									
In-person	323	1.0 (-1.0–3.0)	**< 0.01**	3.0 (-1.0–7.0)	**< 0.01**	1.0 (-1.0–4.0)	**< 0.01**	5.0 (-1.0–12.0)	**< 0.01**
Online	28	0.5 (-2.0–3.0)	0.42	2.0 (-1.0–4.5)	**0.08**	2.0 (-1.0–3.0)	**0.01**	4.5 (-1.5–9.5)	**0.05**

All p-values for non-parametric data were calculated using Wilcoxon Signed Rank Test. Median: median difference

* IQR: *interquartile range

*P *< .05 was considered statistically significant. *P* in bold means the result is statistically significant

Mean difference and 95% CI with associated *p*-value was reported for the variable*

## Discussion

This study demonstrated that participation in the CWBW program led to significant improvements in cooking confidence, adherence to the MD principles, and engagement with the MFHL. These improvements were not only observed immediately after the intervention but were sustained for up to 12 months for MD adherence and cooking confidence, underscoring the durability of the program’s impact.

The reported dietary changes, including increased intake of vegetables, fruits, nuts, fish, and olive oil, along with reduced consumption of grain, mirror results from other nutrition and culinary education interventions. Prior studies have shown that providing individuals with both knowledge and practical cooking skills supports healthier food choices and fosters long-term improvements in dietary behavior. For instance, our findings concur with results from a randomized controlled trial (RCT) that found participants receiving culinary education were more likely (Odds Ratio = 2.93; 95% CI 1.73, 4.95) to adhere to the MD [25]. A study by Morelli et al. also reported a statistically significant increase in participants’ adherence to the MD (T0: 6.03 ± 2.33 vs. T1: 6.96 ± 2.03, *P =* .002) [26]. The consistency of our findings with those reported in previous studies suggests that when people have the knowledge and skills to cook healthily, they will eat more nutritious foods and less processed foods. These findings indicate the benefits of a nutrition education program in improving healthy dietary habits. Our results contribute to this literature by showing that improvements were evident across diverse demographic groups, underscoring the potential for broad scalability of CWBW.

One of the key objectives of nutrition education and cooking classes is to build cooking confidence [27]. Cooking confidence refers to the ability to measure ingredients, follow recipes, use fresh foods, and feel comfortable with basic techniques [28, 29]. We observed a significant increase in cooking skills confidence. Specifically, more participants reported being very confident following a simple recipe, cooking from basic ingredients, preparing new foods, substituting ingredients, handling knives safely, and using various cooking techniques. These findings align with those from previous studies [30, 31]. For example, the PRx + Cooking program reported a mean increase in cooking skills confidence scores from baseline to follow-up, a mean score changes of 0.6 ± 0.5 (*P* < .001) [30]. Similarly, a vegetable cooking skills program in Minneapolis-St. Paul also reported an increase in the mean score from 4.0 ± 1.0 before the course to 4.4 ± 0.7 after the course (*P* < .001) . These findings demonstrate that hands-on cooking education builds skills that help individuals make healthier food choices and rely less on processed foods. Increased cooking confidence is especially important because it is associated with better diet quality and long-term health.

We observed an increase in MFHL scores, in particular, in making smart food choices, increasing physical activity, managing stress, and providing relationship support. The MFHL framework sets this program apart by addressing health holistically, which may contribute to the program’s effectiveness and the sustained improvements observed. Our results suggest that framing dietary behavior within a broader lifestyle context may enhance the effectiveness of interventions and promote sustained improvements in health.

Subgroup analyses further highlighted that the program was beneficial across diverse populations, including individuals of different genders, marital statuses, ages, races, educational levels, and income. More specifically, younger participants, women, and those with lower to moderate incomes showed particularly increased median scores. Our findings suggest that CWBW may be especially effective for groups at higher risk for diet-related chronic diseases. However, the high level of missing sociodemographic data may have reduced statistical power and introduced selection bias, so our results must be interpreted with caution. The differences in outcomes by gender, particularly the limited improvement in MD adherence among men, warrant further exploration to better tailor future interventions.

### Strengths and limitations

Our study had some strengths. First, the use of validated measures for MD and cooking confidence increased the reliability of findings. Second, follow-up data allowed for an examination of the program’s longer-term impact. However, limitations must be acknowledged. First, as participants self-reported the food they ate and their health conditions, there is a possibility of underreporting or overreporting, which may have introduced reporting bias and led to inaccurate conclusions. Second, there is a potential for selection bias. This may have occurred because individuals with an existing interest in healthy eating or cooking were more motivated to participate, thereby limiting the generalizability of the findings. Third, missing sociodemographic data due to non-response may have affected the interpretation of the study findings and high attrition rates limited the generalizability of our results. These challenges highlight an important lesson: successful participation and retention in community-based research projects require a comprehensive approach. Key strategies include building and maintaining strong community-researcher relationships, maintaining open communication and transparency throughout all research stages, recognizing and respecting cultural differences, understanding what motivates participants, and communicating study findings to the community [32, 33]. Fourth, the analyzed data came from a quasi-experimental design without a control group, which restricts causal inference, and improvements cannot be definitively attributed to the program alone. Thus, our results must be interpreted with caution.

## Conclusion

The CWBW program significantly improved participants’ adherence to the MD, confidence in healthy cooking, and engagement with the MFHL. These findings suggest that community-based nutrition education programs, particularly when rooted in cultural and faith-based contexts, can effectively promote healthier dietary behaviors and support chronic disease prevention.

## Supplementary Information

Supplementary Material 1.

## Data Availability

The data we analyzed was provided by Church Health, and it’s available from the authors upon reasonable request, and with permission from Church Health (Butch Odom; [odomb@churchhealth.org](mailto: odomb@churchhealth.org) ).

## Abbreviations

MFHL: Model for Healthy Living
MD: Mediterranean Diet
CWBW: Cook Well Be Well
CVDs: Cardiovascular Diseases

## References

[1] Hariharan R, Odjidja EN, Scott D, et al. The dietary inflammatory index, obesity, type 2 diabetes, and cardiovascular risk factors and diseases. Obes Rev. 2022;23(1):e13349. 10.1111/obr.13349. 34708499 10.1111/obr.13349

[2] Ahmad FB, Cisewski JA, Xu J, Anderson RN. Provisional mortality data - United States, 2022. MMWR Morb Mortal Wkly Rep. 2023;72(18):488–92. 37141156 10.15585/mmwr.mm7218a3 PMC10168603

[3] Centers for disease control and prevention. Diagnosed Diabetes: U.S. Diabetes Surveillance System. 2021. https://gis.cdc.gov/grasp/diabetes/diabetesatlas-surveillance.html. Accessed 21 November 2024.

[4] Shelby county health department. 2012–2018 community health improvement plan. Shelby County, TN. 2015. https://shelbytnhealth.com/Archive/ViewFile/Item/54. Accessed 21 November 2024.

[5] U.S. Department of Agriculture and U.S. Department of health and human services. Dietary guidelines for Americans, 2020–2025. 9th Edition. December 2020. Available at https://www.dietaryguidelines.gov. Accessed 10 October 2024.

[6] Yin W, Löf M, Chen R, Hultman CM, Fang F, Sandin S. Mediterranean diet and depression: a population-based cohort study. Int J Behav Nutr Phys Act. 2021;18(1):153. 34838037 10.1186/s12966-021-01227-3 PMC8627099

[7] Damigou E, Faka A, Kouvari M, Anastasiou C, Kosti RI, Chalkias C, Panagiotakos D. Adherence to a Mediterranean type of diet in the world: a geographical analysis based on a systematic review of 57 studies with 1,125,560 participants. Int J Food Sci Nutr. 2023;74:799–813. 37771002 10.1080/09637486.2023.2262781

[8] Ozsoy S, Sultanoglu N, Sanlidag T. The role of Mediterranean diet and gut microbiota in type-2 diabetes mellitus associated with obesity (diabesity). J Prev Med Hyg. 2022;63(Suppl 3):E87–92. 36479504 10.15167/2421-4248/jpmh2022.63.2S3.2751 PMC9710419

[9] Milenkovic T, Bozhinovska N, Macut D, Bjekic-Macut J, Rahelic D, Velija Asimi Z, Burekovic A. Mediterranean diet and type 2 diabetes mellitus: a perpetual inspiration for the scientific world. Rev Nutrients. 2021;13:1307. 10.3390/nu13041307 PMC8071242 33920947

[10] Suga H. Household food unavailability due to financial constraints affects the nutrient intake of children. Eur J Public Health. 2019;29(5):816–20. 10.1093/eurpub/cky263. 30561601 10.1093/eurpub/cky263

[11] DelavegaE, Blumenthal MG. 2024 Memphis Poverty Fact Sheet. Memphis, TN: The University of Memphis School of Social Work. 2024. https://www.gmbs-consulting.com/project/memphis-poverty-fact-sheet-2024.

[12] Dexter AS, Pope JF, Erickson D, Fontenot C, Ollendike E, Walker E. Cooking education improves cooking confidence and dietary habits in veterans. Diabetes Educ. 2019;45(4):442–9. 31072223 10.1177/0145721719848429

[13] Fernandes CM, Bernardo GL, Fernandes AC, et al. Impact of a cooking intervention on the cooking skills of adult individuals with type 2 diabetes mellitus: a pilot study. Nutrients. 2024;16(11):1657. 10.3390/nu16111657. Published 2024 May 28. 38892590 10.3390/nu16111657 PMC11175113

[14] Hutchinson J, Watt JF, Strachan EK, Cade JE. Evaluation of the effectiveness of the ministry of food cooking programme on self-reported food consumption and confidence with cooking. Public Health Nutr. 2016;19(18):3417–27. 10.1017/S1368980016001476. 27434464 10.1017/S1368980016001476 PMC10270814

[15] Arslan S, Tari Selcuk K, Sahin N, et al. The relationship between food and cooking skills, and eating behaviors in people with overweight or obesity. Int J Obes. 2023;47:60–6. 10.1038/s41366-022-01238-5. 10.1038/s41366-022-01238-5 36380081

[16] UnitedStates Census Bureau. Memphis city, Tennessee- Census Bureau Profile. Accessed 05 December 2024. https://data.census.gov/profile?g=160XX00US4748000.

[17] Stefler D, Malyutina S, Kubinova R, Pajak A, Peasey A, Pikhart H, Brunner E, Bobak M, et al. Mediterranean diet score and total and cardiovascular mortality in Eastern Europe: the HAPIEE study. Eur J Nutr. 2017;56(1):421–9. 26578528 10.1007/s00394-015-1092-x PMC5290049

[18] Trichopoulou A, Kouris-Blazos A, Wahlqvist ML, et al. Diet and overall survival in elderly people. BMJ. 1995;311(7018):1457–60. 8520331 10.1136/bmj.311.7018.1457 PMC2543726

[19] Barton KL, Wrieden WL, Anderson AS. Validity and reliability of a short questionnaire for assessing the impact of cooking skills interventions. J Hum Nutr Diet. 2011;24(6):588–95. 21649746 10.1111/j.1365-277X.2011.01180.x

[20] Ottawa:Ottawa Public Health, Plan, Shop, Cook, Enjoy! 2014 adapted from Kingston, Frontenac and Lennox & Addington (KFL&A) Public Health 2014 Annual Report. 2014. https://www.kflaph.ca/en/about-us/annual-reports.aspx.

[21] Pelletier JE, Laska MN. Balancing healthy meals and busy lives: associations between work, school, and family responsibilities and perceived time constraints among young adults. J Nutr Educ Behav. 2012;44(6):481–9. 10.1016/j.jneb.2012.04.001. Epub 2012 Sep 25. PMID: 23017891; PMCID: PMC3496024. 10.1016/j.jneb.2012.04.001 PMC3496024 23017891

[22] Welch N, McNaughton SA, Hunter W, Hume C, Crawford D. Is the perception of time pressure a barrier to healthy eating and physical activity among women? Public Health Nutr. 2009;12(7):888–95. Epub 2008 Jul 23. PMID: 18647424. 18647424 10.1017/S1368980008003066

[23] Estrada E, Ferrer E, Pardo A. Statistics for evaluating pre-post change: relation between change in the distribution center and change in the individual scores. Front Psychol. 2019;9:2696. 10.3389/fpsyg.2018.02696. 30671008 10.3389/fpsyg.2018.02696 PMC6331475

[24] Brydges CR. Effect size guidelines, sample size calculations, and statistical power in gerontology. Innov Aging. 2019;3(4):igz036. 10.1093/geroni/igz036. Published 2019 Sep 4. 31528719 10.1093/geroni/igz036 PMC6736231

[25] Razavi AC, Sapin A, Monlezun DJ, et al. Effect of culinary education curriculum on Mediterranean diet adherence and food cost savings in families: a randomized controlled trial. Public Health Nutr. 2021;24(8):2297–303. 32744215 10.1017/S1368980020002256 PMC10195617

[26] Morelli C, Avolio E, Galluccio A, et al. Nutrition education program and physical activity improve the adherence to the Mediterranean diet: impact on inflammatory biomarker levels in healthy adolescents from the DIMENU longitudinal study. Front Nutr. 2021;8:685247. 10.3389/fnut.2021.685247. 34350206 10.3389/fnut.2021.685247 PMC8326330

[27] Garcia AL, Reardon R, McDonald M, Vargas-Garcia EJ. Community interventions to improve cooking skills and their effects on confidence and eating behaviour. Curr Nutr Rep. 2016;5(4):315–22. 27882266 10.1007/s13668-016-0185-3 PMC5097072

[28] Renard M, Kelly DT, Ní Chéilleachair N, Lavelle F, Ó Catháin C. Cooking and food skills confidence of team sport athletes in Ireland. Nutr Bull. 2023;48(3):329–42. 10.1111/nbu.12625. 37435875 10.1111/nbu.12625

[29] Martins CA, Machado PP, Louzada MLDC, Levy RB, Monteiro CA. Parents’ cooking skills confidence reduce children’s consumption of ultra-processed foods. Appetite. 2020;144:104452. 10.1016/j.appet.2019.104452. 31521769 10.1016/j.appet.2019.104452

[30] Ylitalo KR, Janda KM, Clavon R, et al. Cross-sector partnerships for improved cooking skills, dietary behaviors, and belonging: findings from a produce prescription and cooking education pilot program at a federally qualified health center. Nutrients. 2023;15(19):4098. 10.3390/nu15194098. 37836383 10.3390/nu15194098 PMC10574603

[31] Overcash F, Ritter A, Mann T, et al. Impacts of a vegetable cooking skills program among low-income parents and children. J Nutr Educ Behav. 2018;50(8):795–802. 10.1016/j.jneb.2017.10.016. 29242140 10.1016/j.jneb.2017.10.016

[32] Tran TPT, Cormier JC, Hopwood CA, Foster J, Scheib I, Li F, et al. Building trust for community-engaged research: recommendations from a qualitative study. J Participatory Res Methods. 2025;6(2):89–113. 10.35844/001c.131692.

[33] Saleh A, Saelens B, Hayes M, Health equity community advisory committee, coker TR. Community partnership guide for engaging with academic researchers. Prog Community Health Partnersh. 2022;16(1):129–34. 10.1353/cpr.2022.0012. 35342117 10.1353/cpr.2022.0012 PMC9148913

