# Supplementary material for: Evaluation of the effectiveness of nutrition education program in promoting healthy dietary habits in Memphis, Tennessee: a quasi-experimental pilot study
Source: BMC Public Health. 2026 Apr 14;26:1669. doi: 10.1186/s12889-026-27392-3 (PMC13195994; doi:10.1186/s12889-026-27392-3)
Supplement: Supplementary file 1 — Supplementary Material 1. [file 12889_2026_27392_MOESM1_ESM.docx]

SUPPLEMENTAL MATERIALS

Supplemental Material 1

Cook Well Be Well Nutrition Education and Cooking class program

Informed Consent Statement

Church Health is committed to ensuring Cook Well Be Well Cooking Classes result in improved health outcomes for participants. We use the survey information to determine how well we are doing in that pursuit. By completing this survey, you are indicating that you consent to our use of this information for program improvement purposes. We will never use or share any of your personal or identifying information.

Date

**Q1 Are you a Church Health Patient?**

- No
- Yes

**Q2 How did you hear about Cook Well Be Well?**

- Social Media (Facebook, Instagram, etc.)
- Other, Please Specify:
- Church Health Staff
- Church Health Website
- Church/Faith Community. If so, please type in name of church below:
- Email
- Well App

**Q4 How did you hear about CWBW church?**

________________________________________________________________

**Q5 How did you hear about CWBW?**

________________________________________________________________

**Q6 Zip Code                       City                               State**

________________________________________________________________

**Q7 Age**

- <18
- 18-24
- 25-34
- 35-44
- 45-54
- 54-65
- >65

**Q8 Gender**

- Male
- Female

**Q9 Marital Status**

- Married
- Widowed
- Divorced
- Separated
- Single/Never married

**Q10 Race**

- Black or African American
- White or Caucasian
- American Indian/Native American or Alaska Native
- Asian
- Native Hawaiian or other Pacific Islander
- Other
- Prefer Not to say

**Q11 Spanish/Hispanic Descent**

- Yes
- No

**Q12 Household Income**

- < $5,000
- $5,001-$10,000
- $10,001-$15,000
- $15,001-$20,000
- $20,001-$30,000
- $30,001-$40,000
- $40,001-$50,000
- $50,001-$70,000
- $70,001-$100,000
- >$100,000

**Q16 Education**

- Got a 4 year college degree (Bachelor's degree)
- High school diploma, GED, or trade school certificate
- Got a graduate school or professional school degree
- Had some college or vocational school
- I did not finish high school
- Got a 2 year college degree (Associate's Degree)

**Q17 Work Status**

- Employed, full time
- Employed, part time
- Retired
- Not employed, looking for work
- Not employed, NOT looking for work
- Disabled, unable to work

**Q18 Conditions**

- High Blood Pressure
- Diabetes
- Other Chronic Diseases
- None of these

**Q20 Other Chronic Diseases**

________________________________________________________________

**MEDITERRENEAN DIET**

**Q21 How many cups of vegetables do you consume each day?**

- Less than 0.5 cups/day
- 0.5 - 1 cup/day
- Greater than 1 cup/day

**Q22 How many servings of legumes do you consume per week?**

- Less than 1 cup/week
- 1 - 2 cups/week
- Greater than 2 cups/week

**Q23 How many cups of fruit and nuts do you consume per day?**

- Less than 0.5 cups/day
- 0.5 - 1 cup/day
- Greater than 1 cup/day

**Q24 How many ounces of cereal/grains do you consume per day?**

- Less than 5 ounces/day
- 5-7 ounces/day
- Greater than 7 ounces/day

**Q25 How many servings of fish do you consume per week?**

- Less than 1 serving/week
- 1 - 2 servings/week
- Greater than 2 servings/week

**Q26 How many cups of dairy do you consume per day?**

- Less than 1 cup/day
- 1 cup /day
- Greater than 1 cup/day

**Q27 How many servings of meat do you consume per day?**

- Less than 1 servings/day
- 1 serving/day
- Greater than 1 serving/day

**Q28 How many alcohol drinks do you consume per day?**

- Less than 1 drink/day
- 1 - 2 drinks/day
- Greater than 2 drinks/day

**Q29 Do you use olive oil for cooking?**

- No
- Yes

**COOKING PRACTICES/SKILLS**

**Q30 How often do you cook convenience foods and ready-meals?**

- Rarely
- Sometimes
- Most days

**Q31 How often do you put together ready-made ingredients to make a complete meal?**

- Most days
- Sometimes
- Rarely

**Q32 How confident do you feel about being able to cook from basic ingredients?**

- Very confident
- Somewhat confident
- Not at all confident

**Q35 How confident do you feel about following a simple recipe?**

- Very confident
- Somewhat confident
- Not at all confident

**Q36 How confident do you feel about tasting foods that you have not eaten before?**

- Very confident
- Somewhat confident
- Not at all confident

**Q37 How confident do you feel about preparing and cooking foods you have not eaten before?**

- Very confident
- Somewhat confident
- Not at all confident

**Q38 I know how to ...Make substitutions for items in a recipe**

- No
- Sometimes
- Yes

**Q39 I know how to ...Use spices or herbs to flavor food**

- No
- Sometimes
- Yes

**Q40 I know how to ...Handle a knife safely to prepare food?**

- No
- Sometimes
- Yes

**Q41 I know how to ...Use beans, chickpeas, and other meat alternatives in a meat-based recipe?**

- No
- Sometimes
- Yes

**Q42 I know how to....Use leftovers in meals**

- Yes
- Sometimes
- No

**MODEL FOR HEALTHY LIVING**

**Q43 FAITH LIFE: How often are you making an effort in building a relationship with God, your neighbors and yourself?**

- Rarely
- Some days
- Most days

**Q44 MOVEMENT: How frequently do you engage in physical activity each week?**

- 0 - 2 days
- 3 - 5 days
- 5 - 7 days

**Q45 MEDICAL: Is it your practice to partner with and follow healthcare provider advice to manage your medical care?**

- Rarely
- Sometimes
- Most of the time

**Q46 WORK: How often do you feel your skills, talents, and gifts are used within the work environment?**

- Monthly
- Weekly
- Daily

**Q47 EMOTIONAL: Do you feel that you are effectively managing stress and understanding your feelings?**

- Rarely
- Some days
- Most days

**Q48 NUTRITION: How frequently do you make smart food choices during the week?**

- 0 - 2 days
- 3 - 5 days
- 5 - 7 days

**Q49 FRIENDS AND FAMILY: How often are you receiving support through relationships?**

- Rarely
- Some days
- Most days

**Q50 Have you met with the Church Health Registered Dietitian?**

- No
- Yes

**Q51 Have you met with the Church Health - Health Coach?**

- No
- Yes

**Table S1.** Scoring system of food components and questionnaire validation

|  | **Scoring System** | | |  |  |
| --- | --- | --- | --- | --- | --- |
| **Mediterranean Diet** **(max points = 18)** | **0** | **1** | **2** | **Validated** | **References** |
| How many cups of vegetables do you consume each day? Serving size: 1 cup raw leafy greens; 1 cup raw vegetables; 1/2 cup cooked vegetables | <0.5 cups/day | 0.5-1 cup/day | >1 cup/day | Yes | [17,18] |
| How many servings of legumes do you consume per week? Serving size: 1/2 cup cooked beans, peas or lentils | <1 cup/week | 1-2 cups/week | >2 cups/week | Yes |  |
| How many cups of fruit and nuts do you consume per day? Serving size 1 cup combined. Fruit: 3/4 cups per day Nuts: 1/4 cup raw nuts or 2 tablespoons nut butter per day | <0.5 cups/day | 0.5-1cup/day | >1 cup /day | Yes |  |
| How many ounces of cereal/grains do you consume per day? Serving size: 1 oz equivalent is 1 slice of bread, 1 cup of ready-to-eat cereal, or ½ cup of cooked rice, cooked pasta, or cooked cereal. | <5 ounces/day | 5-7 ounces/day | >7 ounces/day | Yes |  |
| How many servings of fish do you consume per week? Serving size: 4 oz. fish fillet; 3 oz. canned tuna | < 1 serving/week | 1-2 servings/week | > 2 servings/week | Yes |  |
| How many cups of dairy do you consume per day? Serving size: 1 cup low-fat milk; 1 oz. cheese; 1 cup low-fat plain yogurt | > 1 cup/day | 1 cup /day | < 1 cup/day | Yes |  |
| How many servings of meat do you consume per day? Serving size: 3 oz. cooked chicken breast; 3 oz. cooked pork tenderloin | >1 serving/day | 1 serving/day | <1 serving/day | Yes |  |
| How many alcohol drinks do you consume per day? Serving size: 5 oz. red wine or 12 oz. beer | >2 drinks/day | <1 drink/day | 1-2 drinks/day | Yes |  |
| Do you use olive oil for cooking? | NO | YES |  | Yes |  |
| **Cooking Confidence (max points = 22)** | **0** | **1** | **2** | Yes | [19,20] |
| How often do you cook convenience foods and ready-meals? | Most days | Sometimes | Rarely | Yes |  |
| How often do you put together ready-made ingredients to make a complete meal? | Most days | Sometimes | Rarely | Yes |  |
| How confident do you feel about being able to cook from basic ingredients? | Not at all confident | Somewhat Confident | Very Confident | Yes |  |
| How confident do you feel about following a simple recipe? | Not at all confident | Somewhat Confident | Very Confident | Yes |  |
| How confident do you feel about tasting foods that you have not eaten before? | Not at all confident | Somewhat Confident | Very Confident | Yes |  |
| How confident do you feel about preparing and cooking foods you have not eaten before? | Not at all confident | Somewhat Confident | Very Confident | Yes |  |
| I know how to ...Make substitutions for items in a recipe | NO | Sometimes | YES | Yes |  |
| I know how to ...Use spices or herbs to flavor food | NO | Sometimes | YES | Yes |  |
| I know how to ...Handle a knife safely to prepare food (like cutting an onion) | NO | Sometimes | YES | Yes |  |
| I know how to ...Use beans, chickpeas, and other meat alternatives in a meal once a week | NO | Sometimes | YES | Yes |  |
| I know how to....Use leftovers in meals | NO | Sometimes | YES | Yes |  |
| **Model for Healthy Living (max points = 14 points)** | **0** | **1** | **2** |  | [21,22] |
| FAITH LIFE: How often are you making an effort in building a relationship with God, your neighbors, and yourself? | Rarely | Some Days | Most Days | No |  |
| MOVEMENT: How frequently do you engage in physical activity each week? | 0-2 days | 3-5 days | 5-7 days | No |  |
| MEDICAL: Is it your practice to partner with and follow healthcare provider advice to manage your medical care? | Rarely | Sometimes | Most of the time | No |  |
| WORK: How often do you feel your skills, talents, and gifts are used within the work environment? | Daily | Weekly | Monthly | No |  |
| EMOTIONAL: Do you feel that you are effectively managing stress and understanding your feelings? | Rarely | Some Days | Most Days | No |  |
| NUTRITION: How frequently do you make smart food choices during the week? | 0-2 days | 3-5 days | 5-7 days | No |  |
| FRIENDS AND FAMILY: How often are you receiving support through relationships? | Rarely | Some Days | Most Days | No |  |

**Table S2.** Effect of CWBW cooking class program on participants MD habits (n =351)

| Mediterranean dietary practices | Responses | Presurvey n (%) | Immediate Post n (%) | *P* |
| --- | --- | --- | --- | --- |
| Cups of vegetables consumed per day | <0.5 cups/day | 126 (36) | 59 (17) | **<.01** |
|  | 0.5-1 cups/day | 162 (45) | 163 (46) |  |
|  | > 1 cup/ day | 64 (18) | 129 (37) |  |
| Cups of fruits and nuts consumed per day |  |  |  | **.01** |
|  | <0.5 cups/day | 133 (38) | 97 (28) |  |
|  | 0.5-1 cups/day | 153 (44) | 157 (44) |  |
|  | > 1 cup/ day | 65 (18) | 97 (28) |  |
| Cups of dairy per day |  |  |  | .69 |
|  | <1 cup/day | 164 (47) | 171 (49) |  |
|  | 1 cup/day | 115 (33) | 106 (30) |  |
|  | > 1 cup/ day | 72 (20) | 74 (21) |  |
| Servings of legumes per week |  |  |  | .14 |
|  | <1 cup/week | 184 (52) | 156 (44) |  |
|  | 1-2 cups/week | 124 (35) | 140 (40) |  |
|  | >2 cups/week | 43 (12) | 55 (16) |  |
| Ounces of cereal/grains per day |  |  |  | **<.01** |
|  | <5 ounces/day | 182 (52) | 160 (46) |  |
|  | 5-7 ounces/day | 30 (9) | 146 (41) |  |
|  | >7 ounces/day | 139 (40) | 45 (13) |  |
| Servings of fish per week |  |  |  | **.01** |
|  | < 1 serving/week | 194 (55) | 149 (42) |  |
|  | 1-2 servings/week | 133 (38) | 163 (46) |  |
|  | > 2 servings/week | 24 (7) | 39 (11) |  |
| Servings of meat per day |  |  |  | .64 |
|  | <1 serving/day | 63 (18) | 72 (20) |  |
|  | 1 serving/day | 149 (42) | 136 (39) |  |
|  | >1 serving/day | 139 (40) | 143 (41) |  |
| Alcohol drinks per day |  |  |  | .18 |
|  | <1 drink/day | 315 (90) | 306 (87) |  |
|  | 1-2 drinks/day | 23 (6) | 36 (10) |  |
|  | >2 drinks/day | 13 (4) | 9 (3) |  |
| Do you use olive oil for cooking? |  |  |  | **<.01** |
|  | Yes | 265 (75) | 309 (88) |  |
|  | No | 86 (25) | 42 (12) |  |

**Notes:** McNemar-Bowker Test was used to determine if there was a statistically significant proportional difference from pre- to immediate post-intervention. A *P <* 0.05 was considered statistically significant.

**Table S3.** Effect of CWBW cooking class program on participants cooking confidence/practices (n =351)

| Cooking Confidence/Practices | Responses | Presurvey  n (%) | Immediate Post n (%) | *P* |
| --- | --- | --- | --- | --- |
| Cook convenience foods and ready meals | Rarely | 158 (45) | 183 (52) | **.01** |
|  | Sometimes | 124 (35) | 131 (37) |  |
|  | Most days | 70 (20) | 37 (11) |  |
| Put together ready-made ingredients to make a complete meal? |  |  |  | .09 |
|  | Rarely | 101 (29) | 92 (26) |  |
|  | Sometimes | 156 (44) | 188 (54) |  |
|  | Most days | 94 (27) | 71 (20) |  |
| Confidence in cooking from basic ingredients |  |  |  | **<.01** |
|  | Not at all confident | 44 (12) | 10 (3) |  |
|  | Somewhat confident | 139 (40) | 125 (35) |  |
|  | Very confident | 168 (48) | 216 (61) |  |
| Confidence following a simple recipe |  |  |  | **<.01** |
|  | Not at all confident | 19 (5) | 3 (1) |  |
|  | Somewhat confident | 97 (28) | 73 (21) |  |
|  | Very confident | 235 (67) | 275 (78) |  |
| Confidence preparing and cooking new foods. |  |  |  | **.02** |
|  | Not at all confident | 44 (13) | 21 (6) |  |
|  | Somewhat confident | 155 (44) | 169 (48) |  |
|  | Very confident | 152 (43) | 161 (46) |  |
| Confidence in tasting new foods |  |  |  | .06 |
|  | Not at all confident | 31 (9) | 17 (5) |  |
|  | Somewhat confident | 132 (38) | 119 (34) |  |
|  | Very confident | 188 (53) | 215 (61) |  |
| Make substitutions for items in a recipe |  |  |  | **<.01** |
|  | No | 105 (30) | 40 (11) |  |
|  | Sometimes | 184 (52) | 210 (60) |  |
|  | Yes | 62 (18) | 101 (29) |  |
| Use spices or herbs to flavor food |  |  |  | **<.01** |
|  | No | 51 (15) | 16 (5) |  |
|  | Sometimes | 159 (45) | 126 (36) |  |
|  | Yes | 141 (40) | 209 (59) |  |
| Handling a knife safely |  |  |  | **<.01** |
|  | No | 24 (7) | 7 (2) |  |
|  | Sometimes | 80 (23) | 40 (11) |  |
|  | Yes | 247 (70) | 304 (87) |  |
| Beans, chickpeas, and meat alternatives once/ week |  |  |  | **<.01** |
|  | No | 128 (37) | 37 (11) |  |
|  | Sometimes | 113 (32) | 99 (28) |  |
|  | Yes | 110 (31) | 215 (61) |  |
| Use leftovers. |  |  |  | **<.01** |
|  | No | 64 (18) | 18 (5) |  |
|  | Sometimes | 146 (42) | 105 (30) |  |
|  | Yes | 141 (40) | 228 (65) |  |

**Notes:** McNemar-Bowker Test was used to determine if there was a statistically significant proportional difference from pre- to immediate post-intervention. A *P <* 0.05 was considered statistically significant.

**Table S4.** Effect of CWBW cooking class program on participants MFHL (n =351).

| Model for Healthy Living | Responses | Presurvey  n (%) | Immediate Post n (%) | *P* |
| --- | --- | --- | --- | --- |
| Make smart food choices during the week | 0-2 days | 142 (41) | 69 (20) | **<.01** |
|  | 3-5 days | 168 (48) | 203 (58) |  |
|  | 5-7 days | 39 (11) | 69 (22) |  |
|  |  |  |  |  |
| Engaging in physical activity/week | 0-2 days | 177 (51) | 127 (36) | **.01** |
|  | 3-5 days | 117 (34) | 159 (46) |  |
|  | 5-7 days | 55(15) | 63 (18) |  |
|  |  |  |  |  |
| Manage stress and understand feelings | Rarely | 48 (14) | 28 (8) | **.01** |
|  | Some days | 172 (49) | 138 (40) |  |
|  | Most days | 129 (37) | 183 (52) |  |
|  |  |  |  |  |
| Receive support through relationships | Rarely | 65 (19) | 32 (9) | **<.01** |
|  | Some days | 112 (32) | 98 (28) |  |
|  | Most days | 172 (49) | 219 (63) |  |
|  |  |  |  |  |
| Manage your medical care | Rarely | 38 (11) | 16 (4) | **<.01** |
|  | Sometimes | 124 (36) | 76 (22) |  |
|  | Most of the times | 187 (53) | 257 (74) |  |
|  |  |  |  |  |
| Build relationship with God, neighbors, and yourself | Rarely | 30 (9) | 22 (6) | .24 |
|  | Some days | 96 (28) | 80 (23) |  |
|  | Most days | 223 (64) | 247 (71) |  |
| Feel your skills, talents, and gifts are used within the work environment | Daily | 191 (54) | 218 (62) | .08 |
|  | Weekly | 103 (30) | 86 (25) |  |
|  | Monthly | 55 (16) | 45 (13) |  |

**Notes:** McNemar-Bowker Test was used to determine if there was a statistically significant proportional difference from pre- to immediate post-intervention. A *P <* 0.05 was considered statistically significant.
